# Supplementary figures and images for: Newborn Screening for Inborn Errors of Metabolism by Next-Generation Sequencing Combined with Tandem Mass Spectrometry
Source: Int J Neonatal Screen. 2024 Mar 29;10(2):28. doi: 10.3390/ijns10020028 (PMC11036227; doi:10.3390/ijns10020028)

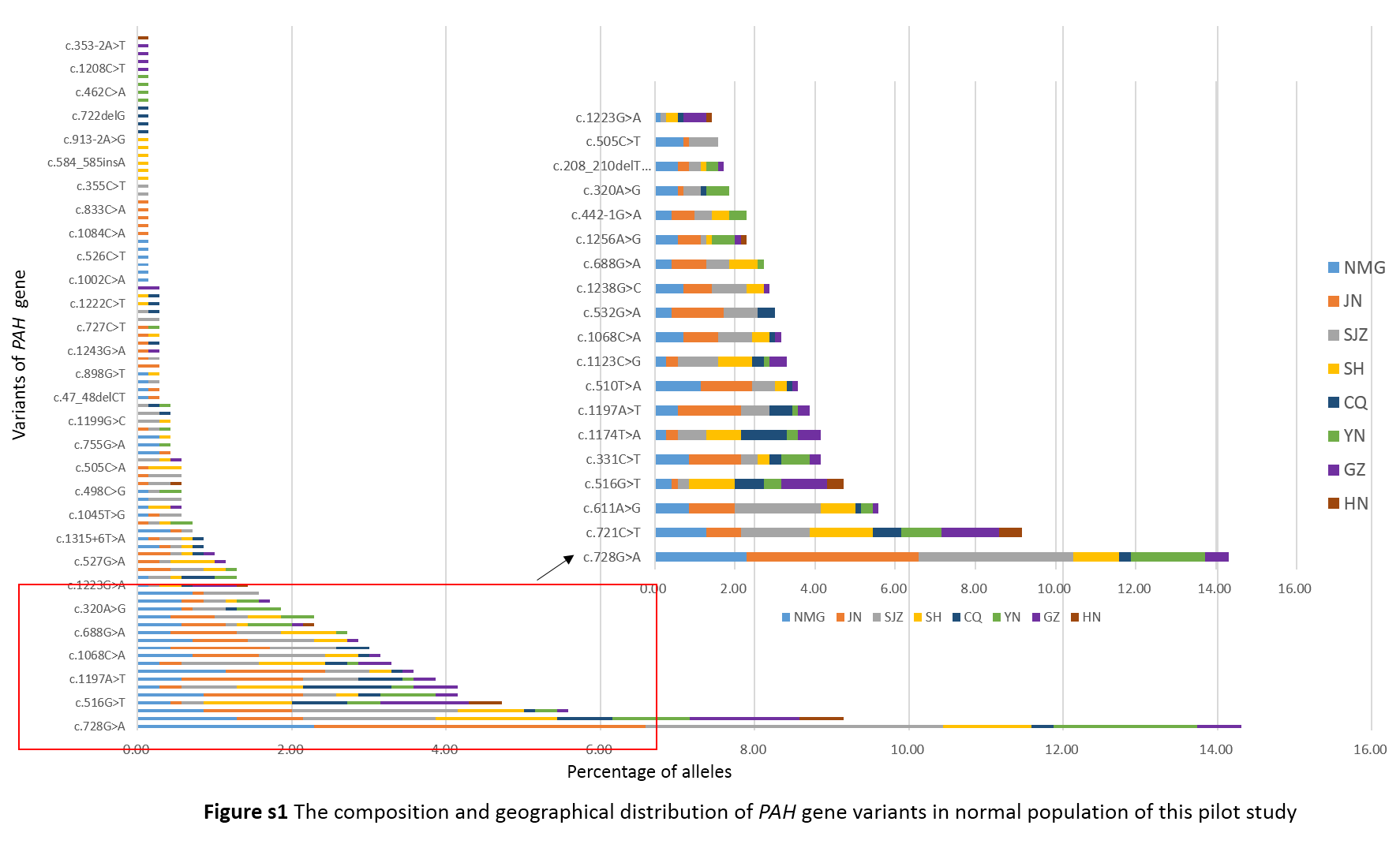

Supplement: Supplementary file 1 [file IJNS-10-00028-s001.zip › Supplemental Figure S1.tif]

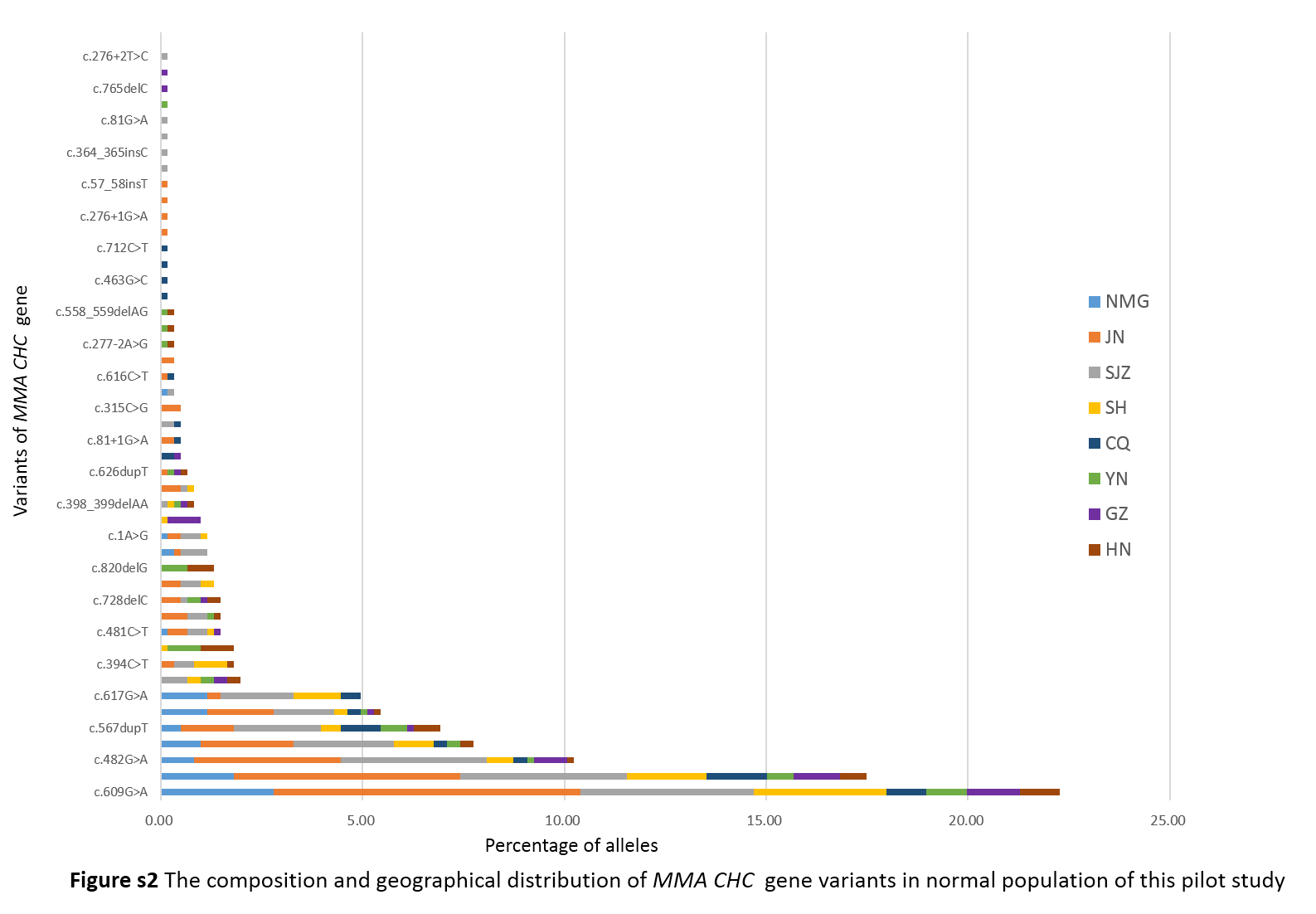

Supplement: Supplementary file 1 [file IJNS-10-00028-s001.zip › Supplemental Figure S2.tif]

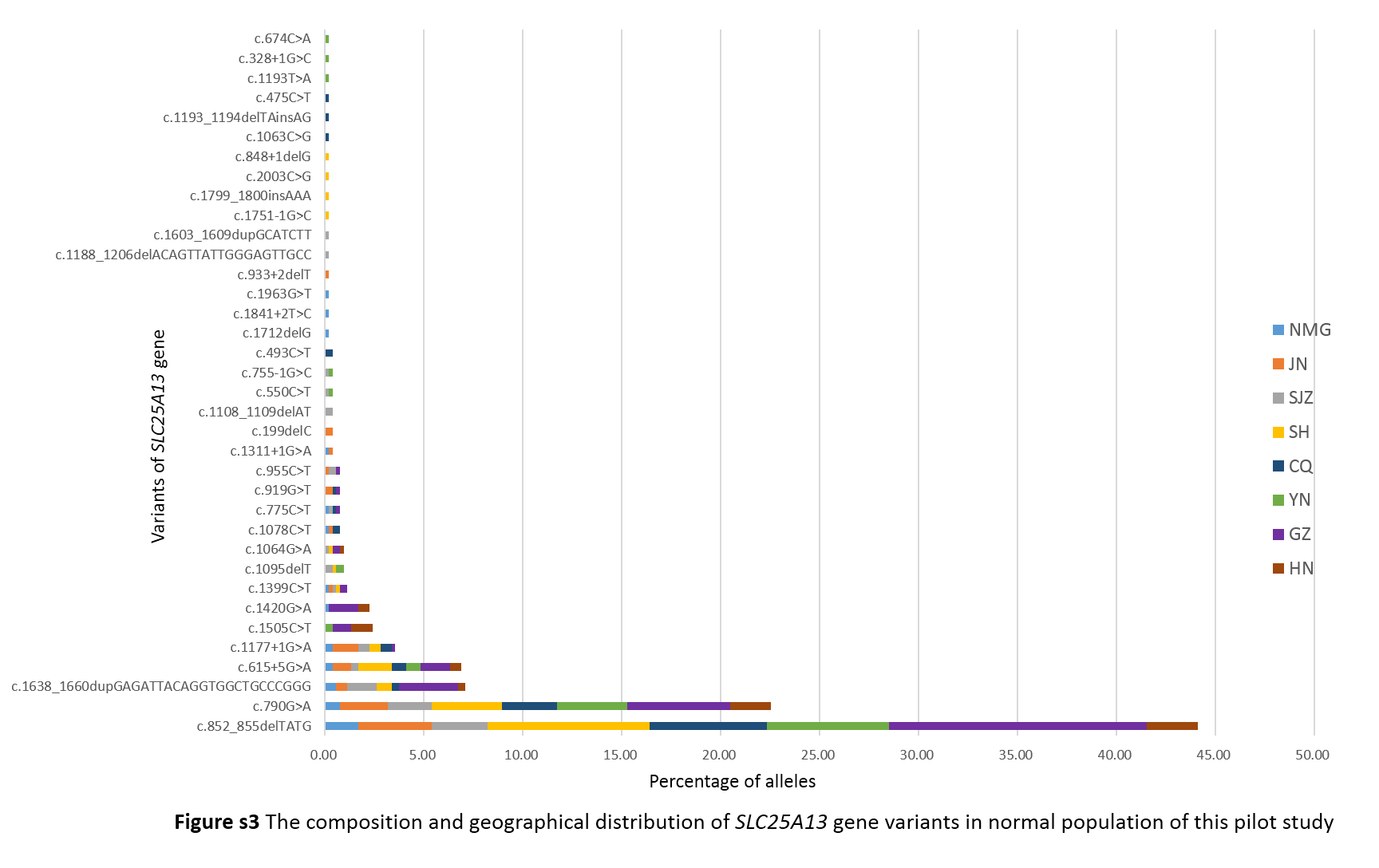

Supplement: Supplementary file 1 [file IJNS-10-00028-s001.zip › Supplemental Figure S3.tif]

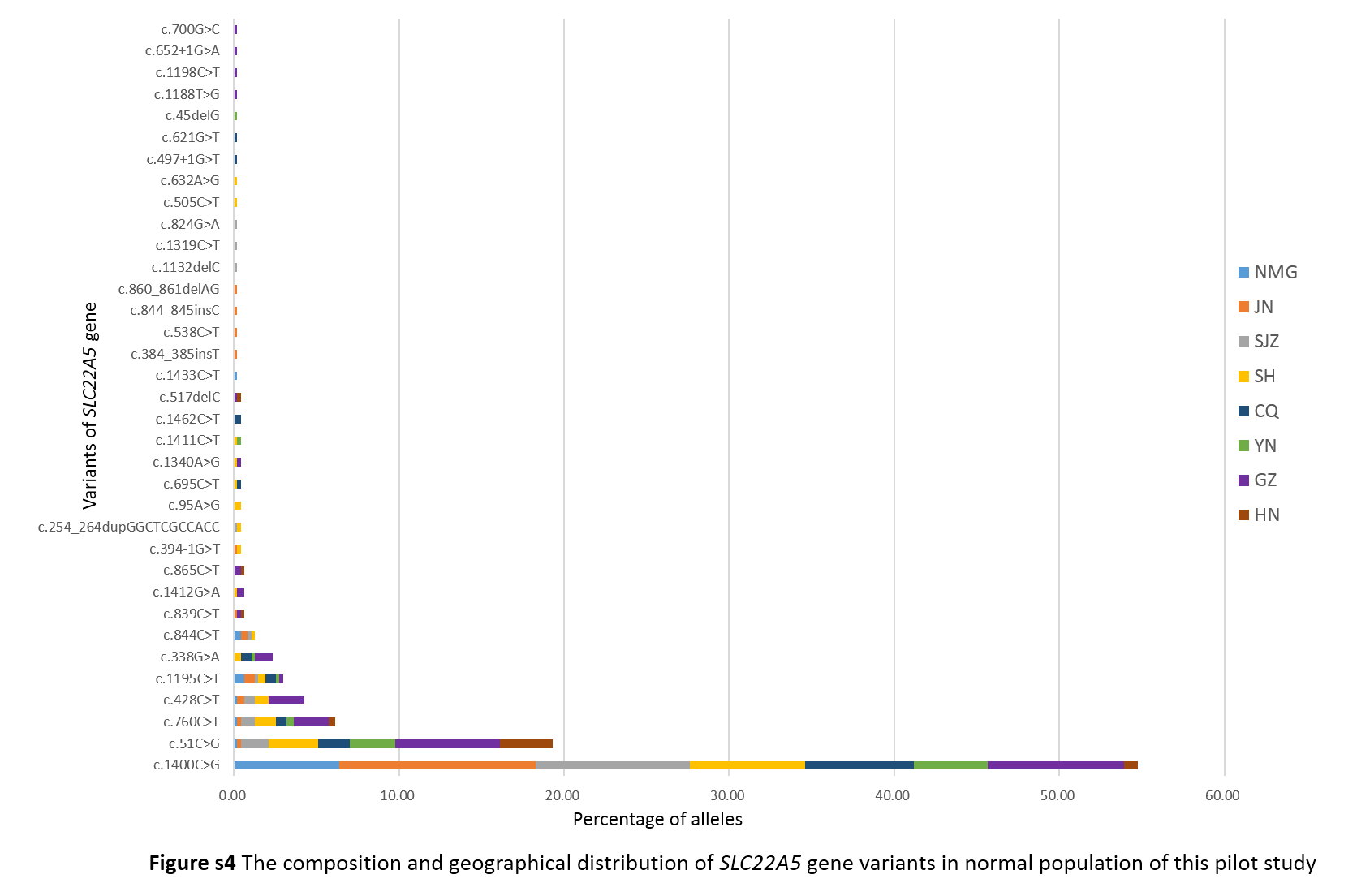

Supplement: Supplementary file 1 [file IJNS-10-00028-s001.zip › Supplemental Figure S4.tif]

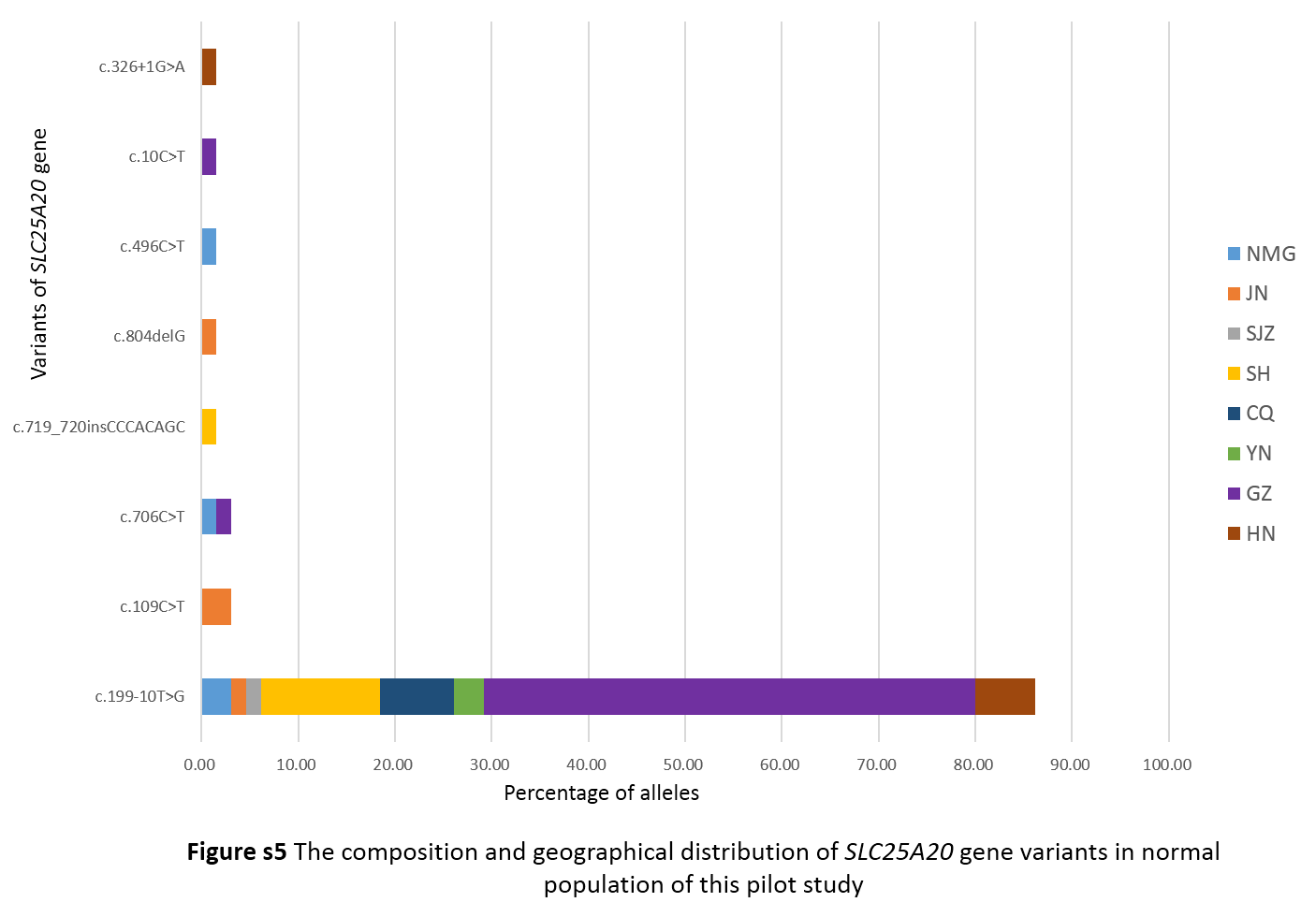

Supplement: Supplementary file 1 [file IJNS-10-00028-s001.zip › Supplemental Figure S5.tif]
